# Effective γδ T‐cell clinical therapies: current limitations and future perspectives for cancer immunotherapy

**DOI:** 10.1002/cti2.1492

**Published:** 2024-02-19

**Authors:** Isabella A Revesz, Paul Joyce, Lisa M Ebert, Clive A Prestidge

**Affiliations:** ^1^ Clinical Health Sciences University of South Australia Adelaide SA Australia; ^2^ Centre for Cancer Biology SA Pathology and University of South Australia Adelaide SA Australia; ^3^ Cancer Clinical Trials Unit Royal Adelaide Hospital Adelaide SA Australia; ^4^ School of Medicine The University of Adelaide Adelaide SA Australia

**Keywords:** bisphosphonates, combination therapy, immunotherapy, lipid nanocarriers, nanomedicine, γδ T cells

## Abstract

γδ T cells are a unique subset of T lymphocytes, exhibiting features of both innate and adaptive immune cells and are involved with cancer immunosurveillance. They present an attractive alternative to conventional T cell‐based immunotherapy due, in large part, to their lack of major histocompatibility (MHC) restriction and ability to secrete high levels of cytokines with well‐known anti‐tumour functions. To date, clinical trials using γδ T cell‐based immunotherapy for a range of haematological and solid cancers have yielded limited success compared with *in vitro* studies. This inability to translate the efficacy of γδ T‐cell therapies from preclinical to clinical trials is attributed to a combination of several factors, e.g. γδ T‐cell agonists that are commonly used to stimulate populations of these cells have limited cellular uptake yet rely on intracellular mechanisms; administered γδ T cells display low levels of tumour‐infiltration; and there is a gap in the understanding of γδ T‐cell inhibitory receptors. This review explores the discrepancy between γδ T‐cell clinical and preclinical performance and offers viable avenues to overcome these obstacles. Using more direct γδ T‐cell agonists, encapsulating these agonists into lipid nanocarriers to improve their pharmacokinetic and pharmacodynamic profiles and the use of combination therapies to overcome checkpoint inhibition and T‐cell exhaustion are ways to bridge the gap between preclinical and clinical success. Given the ability to overcome these limitations, the development of a more targeted γδ T‐cell agonist‐checkpoint blockade combination therapy has the potential for success in clinical trials which has to date remained elusive.

## Graphical abstract

The clinical efficacy of γδ T‐cell immunotherapies is limited by issues with the pharmacodynamic/pharmacokinetic profiles of commonly used agonists, an inability of administered T cells to infiltrate into solid tumours and limited knowledge of γδ T‐cell checkpoint inhibition. This article reviews the current state of γδ T‐cell immunotherapy and offers viable strategies to enhance the clinical efficacy of this promising cancer treatment.

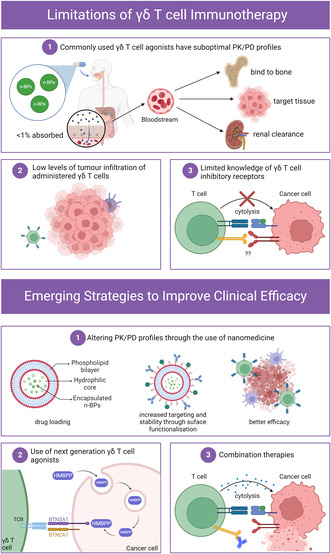

## Introduction

In an aging population, where cancer diagnoses are increasingly prevalent, the pursuit of effective treatments remains a high priority area for biomedical research. Current cancer treatments are typically various combinations of chemotherapy, surgery or radiotherapy; however, these treatments all present challenges with off‐target effects and relapse that can render treatment less effective.^1^ To date, immunotherapy presents one of the more clinically successful forms of treatment and is an ever‐expanding area of research and development. Checkpoint inhibitors (such as programmed cell death protein 1 (PD‐1) or Cytotoxic T‐Lymphocyte Associated protein 4 (CTLA‐4) inhibitors) present one avenue of immunotherapy that aim to circumvent the immunosuppression inherent to many cancers and thus, enable immune cells to carry out their ‘anti‐tumour’ roles.^2^ However, checkpoint inhibitors have only proven effective in certain cancer types, and even in these cohorts, just a fraction of patients experience clinical benefit following treatment.^3^ Alternatively, chimeric antigen receptor T cell (CAR T cell)‐based therapy has been developed in attempts to improve the immune response against malignant cells.^4^ CAR T cells are synthesised by transfection of CAR genes into T cells that are taken either from the patient that will be treated (autologous) or from healthy donors (allogenic).^4^ This approach has proven transformative in the treatment of B‐cell malignancies; however, the manufacturing process is time‐consuming and expensive, and clinical trials for a range of other cancer types (solid tumours in particular) have so far proven underwhelming.^4^, ^5^, ^6^ The limited success with CAR T‐cell treatments in solid tumours is attributed to challenges with tumour infiltration of immune cells, heterogeneous expression of tumour‐associated antigens, immunosuppressive tumour microenvironments (TMEs) as well as CAR‐T‐cell‐related toxicity.^4^, ^6^

γδ T cells are a unique subset of T lymphocytes and offer an attractive option for T cell‐based immunotherapy due to their ability to secrete high levels of cytokines with anti‐tumour functions while displaying features of both innate and adaptive immune cells.^2^, ^7^, ^8^ These features, as well as the ability to recognise a broad range of ligands, have resulted in pre‐clinical and clinical trials using γδ T cells that are well‐tolerated and display minimal off‐target effects, particularly in comparison to traditional T‐cell therapies.^2^ To date, clinical trials using combinations of *in vivo* and *ex vivo* stimulation of γδ T cells have relied on agonists such as aminobisphosphonates (n‐BPs) that have limited bioavailability and have been known to exhibit off‐target toxicity.^2^, ^9^, ^10^, ^11^ Phosphoantigens (pAgs) are bacterial or self‐derived molecules that can more directly stimulate populations of γδ T cells than traditional n‐BPs.^2^, ^12^, ^13^ Unfortunately, pAgs also have limited use as immunotherapeutic agents due to suboptimal pharmacodynamics and kinetics.^14^ The development of new therapeutics that have the capacity to overcome systemic toxicity and immune suppression remains a high priority for cancer research.

This review seeks to highlight and account for the discrepancies between γδ T‐cell immunotherapeutic *in vitro* studies and clinical trials. γδ T‐cell therapy has been shown to be well‐tolerated and potentially presents a way to overcome immune evasion mechanisms often employed by cancerous cells.^15^, ^16^ A high level of tumour‐infiltrating γδ T cells serves as a good indicator of favourable prognostic outcomes, yet thus far, this has not translated into successful clinical trials.^8^ Improving the efficacy of γδ T‐cell immunotherapies is a relatively under‐explored area of research, yet such approaches hold great promise due to the many favourable features that γδ T‐cell therapies display in comparison to conventional αβ T‐cell therapies. Through a better understanding of checkpoint inhibition mechanisms relevant for γδ T cells and improved pharmacodynamics of γδ T‐cell agonists, an ‘off‐the‐shelf’ immunotherapeutic treatment for solid and haematological cancers may be within reach.

## γδ T cells

Tumours develop within unique microenvironments which contain a broad spectrum of cells, soluble factors and bacteria, all of which can contribute to the growth and invasiveness of the tumour. The tumour microenvironment (TME) includes soluble factors such as cytokines and chemokines, as well as stromal, endothelial and immune cells that can function to aid tumour cells in proliferating and invading host tissues.^17^ The immune cells found within the TME are heterogeneous populations, belonging to both the adaptive and innate immune systems, and can elicit either pro or anti‐tumour responses.^18^ Cells of the TME include tumour associated macrophages (TAMs), natural killer cells (NKs) dendritic cells (DCs), myeloid‐derived suppressor cells (MDSCs) and T lymphocytes. Tumour progression and prognosis is largely dependent upon the type of infiltrating immune cells as well as the influence of the TME on activation or suppression of these cells.^17^, ^19^, ^20^

γδ T cells are a subset of T lymphocytes which constitute 1–10% of T cells in the peripheral blood of healthy adults and contribute to populations of tumour‐infiltrating immune cells.^7^ This subset of T cells expresses T‐cell receptors (TCRs) that are composed of γ and δ chains, making them distinct from T cells expressing TCRs composed of α and β chains.^21^, ^22^ While a major proportion of the T cells in the body express αβ TCRs, γδ T cells have garnered interest in recent years due to various features that are unique to this population of lymphocytes that make them ideal targets for cancer immunotherapy.^23^ γδ T cells are unique in that they do not require major histocompatibility complex (MHC) for antigen presentation.^21^ This is advantageous for a number of reasons but especially in immunotherapy, as the efficacy of many traditional T cell‐based immunotherapies is limited by the ability of cancer cells to downregulate MHC class I expression as a means of avoiding immune detection.^24^

In humans, γδ T cells can be categorised according to expression of the δ chain of the TCR and display variable expression of the γ chain of the TCR. There are three main populations of γδ T cells in humans which are classed as Vδ1^+^, Vδ2^+^ and Vδ3^+^ which reside in differing proportions in various tissues.^18^, ^21^, ^25^ Through clonal rearrangement of γ and δ chains, γδ T cells can carry out a diverse range of functions in response to various ligands which has been reviewed extensively elsewhere and many of which remain to be identified.^21^, ^23^, ^26^ In general, the functions of γδ T cells are many and varied and this in part accounts for the difficulty researchers have had in elucidating their specific role within the immune system. γδ T cells link the innate and adaptive immune systems by detecting altered cellular metabolism and are involved in immune surveillance and responses following infection, tissue damage and cellular transformation.^21^ Like other subsets of immune cells, the functions carried out by γδ T cells are largely dependent upon their polarisation which is ultimately influenced by the microenvironment that the cells are exposed to, which will be discussed in detail further on.^7^

**Figure 1:**
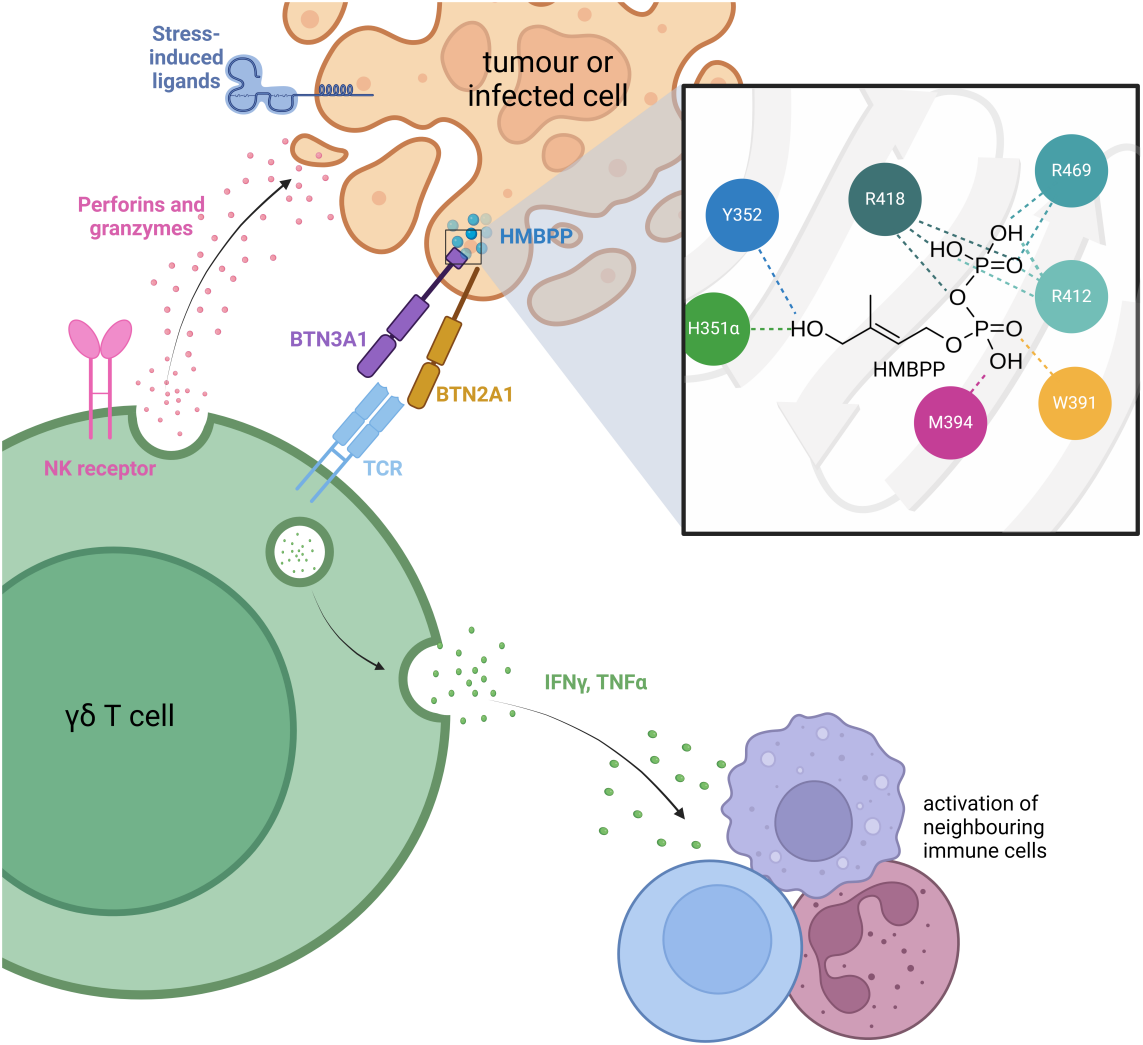
γδ T‐cell‐mediated cytotoxicity against tumour or infected cells. Inset shows HMBPP binding to the binding pocket of the intracellular B30.2 domain of BTN3A1. HMBPP binding induces conformational changes, allowing for the formation of a BTN3A1/BTN2A1 heterodimer which increases affinity for the Vδ2Vγ9 T‐cell receptor (TCR). This induces the release of anti‐inflammatory cytokines such as IFN‐γ and TNF‐α. Natural killer receptors on the surface of Vδ2Vγ9 T cells can also be activated by stress‐induced ligands expressed by target cells, resulting in the release of perforins and granzymes. The figure was created using Biorender, adapted from Mensurado *et al*.^28^

The nature of ligand recognition by γδ T cells remains an area of interest, as the mechanisms involved are not well understood. The difficulty in identifying the molecular mechanisms for the activation of γδ T cells is largely owing to the ability of these lymphocytes to recognise such a diverse range of ligands.^21^ This sits in contrast with conventional αβ T cells, which are limited to the recognition of peptide antigens. Broadly, γδ T cells are considered to be ‘stress sensors’ and can detect altered metabolism within both pathogenic and host cells.^26^ As highlighted in Figure 1, receptors shared by NK cells of the innate immune system are also expressed by γδ T cells such as NKG2D, NKp30 and NKp44, aiding in the detection of stress‐induced ligands.^7^, ^27^ While γδ T cells can recognise a wide range of molecules, which are extensively reviewed elsewhere, pAg‐induced activation of a particular subset of γδ T cells known as Vδ2Vγ9 T cells is currently the best understood mechanism because of its association with cancer immunotherapy.^7^, ^27^, ^28^ However, it is important to note that pAg‐induced activation of Vδ2Vγ9 T cells is not exclusive to cancer and has also been demonstrated in a diverse range of infectious diseases such as tuberculosis, toxoplasmosis and even with SARS‐CoV‐2.^29^

PAgs are naturally occurring intermediates of the mevalonate and non‐mevalonate pathways of isoprenoid biosynthesis and are known to be the main agonists of peripheral blood Vδ2Vγ9 γδ T cells.^21^, ^23^ The primary source of pAgs in humans is the mevalonate pathway, intermediates of which accumulate intracellularly when this pathway is dysregulated such as occurs in hypercholesterolemia and other cardiovascular diseases, and has implications with neurodegenerative diseases such as Alzheimer's and Parkinson's diseases.^21^, ^30^, ^31^ Vδ2Vγ9 T cells are uniquely equipped to recognise foreign and tumour‐derived pAgs without the need for antigen processing or presentation.^17^, ^21^, ^32^ Detection of intracellular pAgs by γδ T cells relies on an ‘inside‐out’ signalling mechanism involving the family of butyrophilin and butyrophilin‐like molecules.^21^, ^23^ Until recently, it was thought that the transmembrane protein BTN3A1 would bind pAgs and directly present them to Vδ2Vγ9 TCRs.^33^ More recently it has been shown that BTN3A1 binds pAgs intracellularly through their B30.2 domain, which likely induces extracellular conformational changes in the transmembrane protein.^34^, ^35^, ^36^ As shown in Figure 1, BTN2A1 has also recently been shown to interact directly with the Vδ2Vγ9 TCR as well as with intracellular pAgs, also through their B30.2 intracellular domain, leading to the hypothesis that a butyrophilin heterodimer forms upon pAg‐induced conformational change and is required for Vδ2Vγ9 TCR binding and activation.^34^, ^37^, ^38^, ^39^ Upon activation by pAgs, Vδ2Vγ9 T cells tend to express a Th_1_‐like phenotype and secrete the proinflammatory cytokines IFN‐γ and TNF‐α.^19^ The cytolytic activity of Vδ2Vγ9 T cells can also be increased by stimulation with pAgs in conjunction with interleukin‐2 (IL‐2) by causing the upregulation of the surface receptor CD16 which upon activation transduces signals that promote Antibody‐Dependent Cellular Cytotoxicity (ADCC) in response to specific antibodies.^23^ These are just some of the ways in which pAg‐induced activation of γδ T cells can induce a variety of effector functions that make them particularly suited for immunosurveillance and killing of malignant or infected cells.

### Role of γδ T cells in cancer

**Figure 2:**
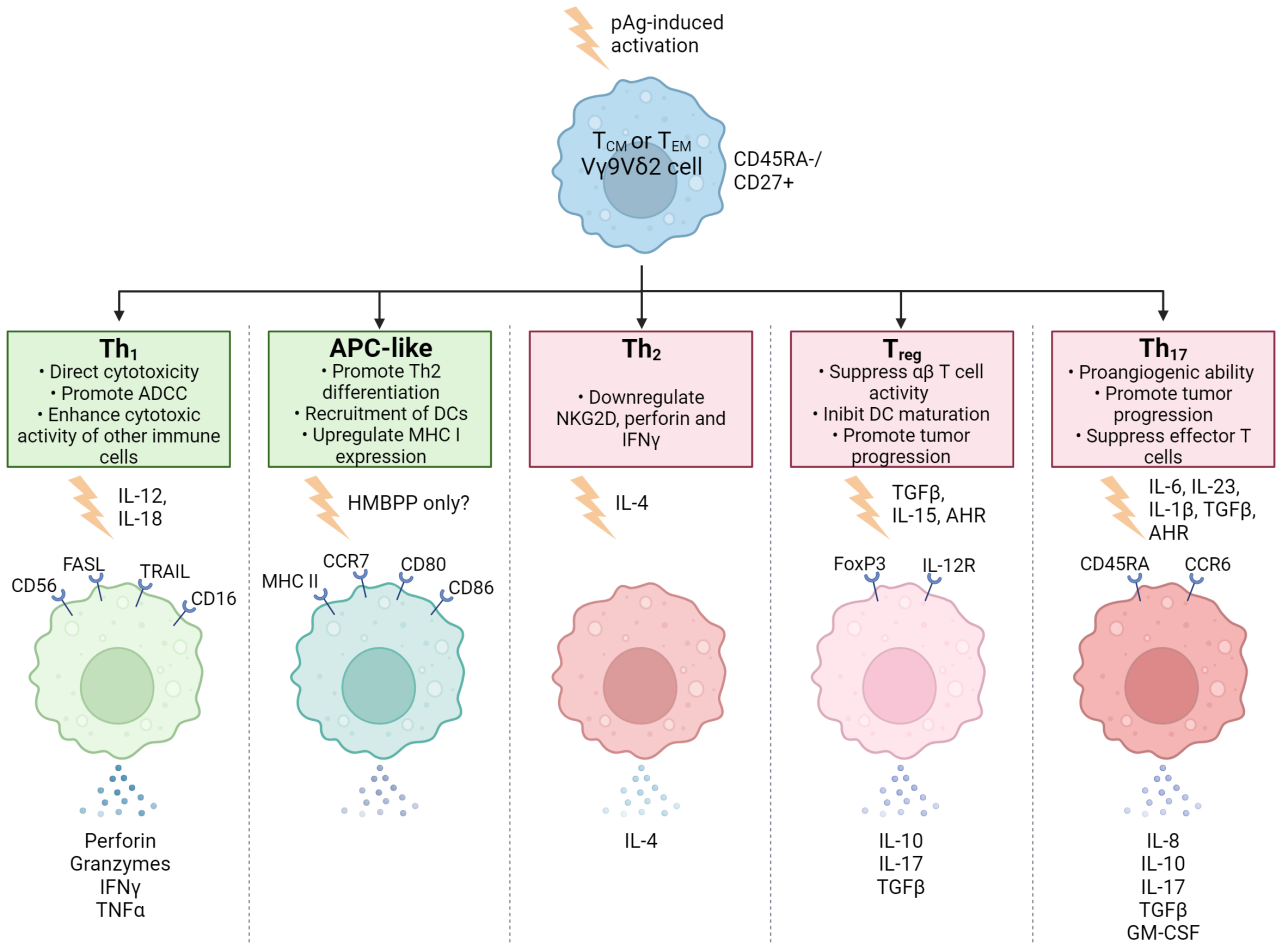
The pro (red) and anti‐tumour (green) roles of Vγ9Vδ2^+^ T cells and the cytokines involved in differentiation into various effector/regulatory functions following pAg‐induced activation.^101^, ^103^ This figure is adapted from Imbert and Olive^8^ and Pang *et al*.,^105^ and was created using Biorender.

The role of γδ T cells in cancer progression remains somewhat controversial due to the plasticity that these cells display. γδ T cells have been detected in many haematological and solid cancers where in some cases, higher levels of circulating and tumour‐infiltrating γδ T cells have been associated with better prognosis while in other cases, the opposite has been suggested.^19^ Given that the primary source of pAgs in humans is *via* the mevalonate pathway, which is often upregulated in transformed cells, it is not surprising that Vδ2Vγ9 T cells are involved in cancer immunosurveillance.^21^, ^40^ Aside from *via* pAgs, Vδ2Vγ9 T cells can also identify tumour cells through interaction with tumour‐associated surface proteins such as F1‐ATPase, NKG2D, TRAIL and CD226 which promote cytolytic activity.^2^, ^21^, ^41^ Vδ2Vγ9 T cells may be attracted to the TME *via* chemokines where they can detect transformed cells; however, few studies to date have investigated the specific receptors expressed by γδ T cells in relation to TME chemokines.^8^ It is thought that the TME is a major factor in the polarisation of tumour‐infiltrating γδ T cells to a pro‐tumour phenotype, thus limiting their effectiveness as cytotoxic cells.^17^, ^19^, ^21^ As observed in Figure 2, Vδ2^+^ cells can differentiate into various functional subsets such as Th_1_, Th_2_, Th_17_ or T_reg_‐like cells, depending on the cytokines that are present within the TME.^19^, ^26^, ^41^, ^42^, ^43^, ^44^

The anti‐tumour role of γδ T cells is relatively well‐established due to the wide range of effector functions that they are known to carry out upon activation. γδ T cells can act through both direct and indirect pathways to kill transformed cells, which has been demonstrated both *in vitro* and *in vivo* in multiple types of cancer.^26^ Higher levels of circulating and tumour‐infiltrating γδ T cells have been correlated with a more favourable prognostic outcome across 25 different solid and haematological cancers, including Acute Myeloid Leukaemia (AML), breast cancer, colon cancer and Burkitt's lymphoma.^8^, ^45^ Cytotoxicity from γδ T cells against transformed cells has been suggested to be largely due to dysregulation of the mevalonate pathway, which is often observed in tumours with p53 mutations, leading to the accumulation of endogenous pAgs.^21^ Another potential source of Vδ2Vγ9‐induced cytotoxicity against cancer cells is hypothesised to be due to the production of pAgs by bacteria within the oncobiome.^23^, ^46^ PAg‐induced activation of γδ T cells results in the secretion of inflammatory cytokines such as IFN‐γ and TNF‐α which have strong anti‐tumour activity, inhibiting angiogenesis as well as inducing DC maturation.^41^, ^47^ However, pAg‐induced activation of Vδ2Vγ9 T cells may result in different outcomes, depending on the cytokine profile of the TME.^41^

In recent years, the pro‐tumour role of γδ T cells has also been realised, although the full extent that these immune cells play in the promotion of tumorigenesis is not fully understood. It is well known that the TME has an abundance of immunosuppressive cytokines such as TGF‐β and IL‐10 which can suppress the normal cytotoxic functions of immune cells.^19^ Figure 2 highlights the polarising effect of these cytokines on γδ T cells, which can cause them to express a T_reg_‐like or Th_17_ phenotype.^19^ When γδ T cells exhibit a T_reg_ or Th_17_ phenotype, they are capable of secreting IL‐17, which has been associated with promotion of tumour cell proliferation and invasion through activation of neutrophils, promoting tumour growth and metastasis.^48^, ^49^, ^50^ The secretion of IL‐8 and GM‐CSF by Th_17_ γδ T cells has been shown to attract immunosuppressive neutrophils to tumour sites.^2^, ^51^ While the pro‐tumour role of γδ T cells is not as well understood as their anti‐tumour role, it is nonetheless an important consideration in the efficacy of γδ T cell‐based immunotherapies.

### Inhibitory receptors

Following immune cell activation, there is a need for mechanisms that prevent uncontrolled activation and proliferation in order to avoid damage to healthy tissues and chronic inflammation.^52^ One such mechanism that immune cells employ is the expression of immune checkpoint receptors.^52^, ^53^ Recently, 3D melanoma models have been used to demonstrate the limited anti‐tumour capacity of tumour‐infiltrating γδ T cells due to increased expression of checkpoint receptors such as PD‐1 and CTLA‐4.^54^ In this study, γδ T‐cell anti‐tumour activity and tumour infiltration were enhanced through the use of checkpoint inhibitors to overcome T‐cell exhaustion.^54^

**Figure 3:**
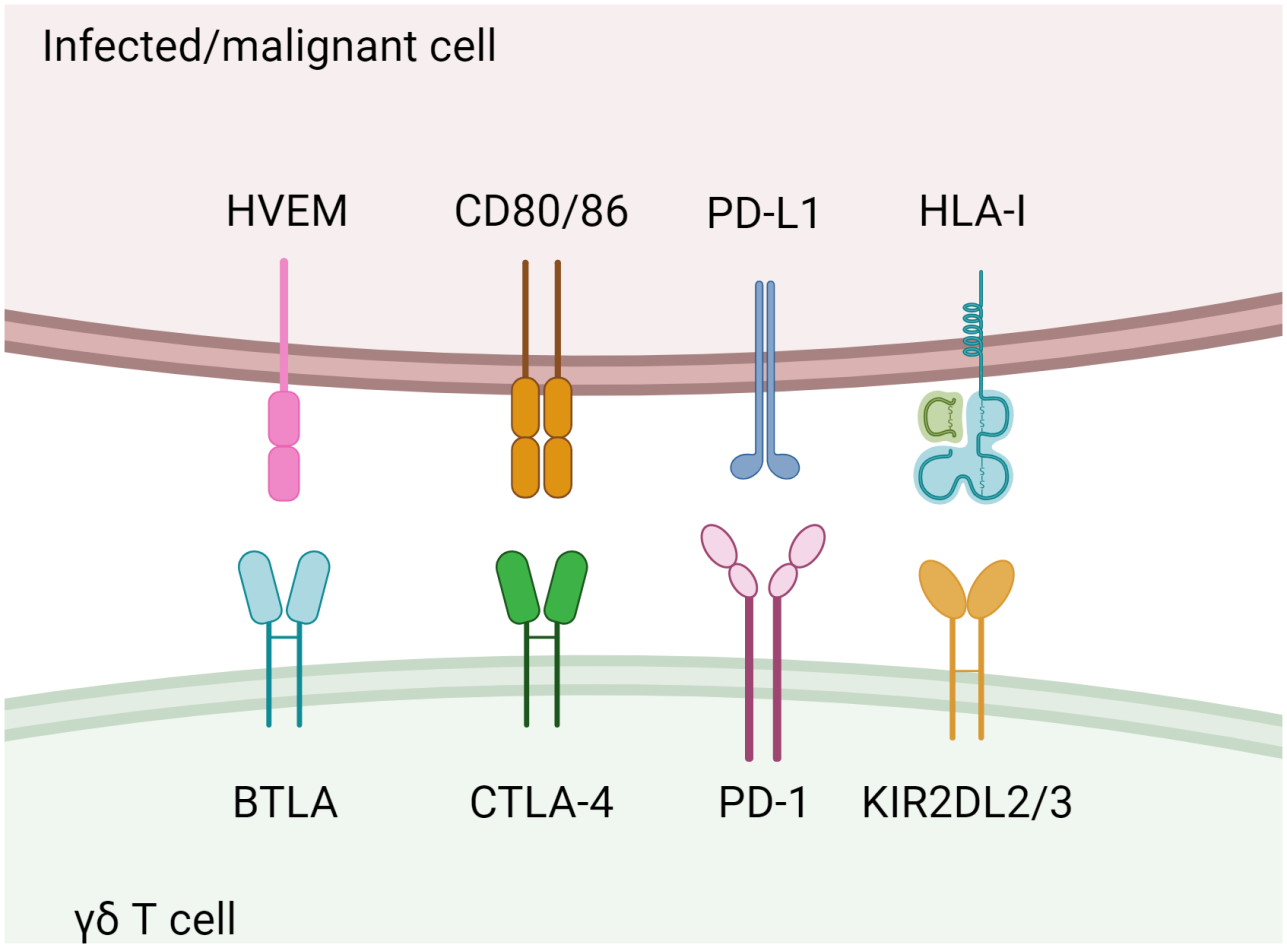
Inhibitory receptors expressed by γδ T cells shown in conjunction with their corresponding ligands. The natural ligand for B and T lymphocyte attenuator (BTLA) is herpesvirus entry mediator (HVEM), CD80/86 expression can inhibit T‐cell activation via cytotoxic T‐lymphocyte‐associated protein 4, programmed cell death protein 1 (PD‐1) is inhibited by programmed cell death ligand 1 (PD‐L1) and killer cell Immunoglobulin‐like receptor (KIR2DL2/3) is associated with human leukocyte antigen I (HLA‐I). This figure is adapted from Gao *et al*.,^106^ and was created using Biorender.

Some progress has been made in recent years in identifying the checkpoint receptors expressed by γδ T cells, some of which are summarised in Figure 3. B and T Lymphocyte Attenuator (BTLA) is an inhibitory receptor expressed by the majority of lymphocytes.^55^ Herpesvirus Entry Mediator (HVEM) is the natural ligand for BTLA and HVEM positive cells have been shown to inhibit Vγ9Vδ2 T‐cell proliferation through interaction with BTLA.^53^, ^56^ In fact, BTLA stimulation of Vγ9Vδ2 T cells may provide a means of immune escape by malignant cells.^53^, ^56^ BTLA has been shown to be upregulated on the surface of γδ T cells in patients with non‐small cell lung cancer, further suggesting the involvement of this molecule in immune evasion by malignant cells.^57^ In a similar way to BTLA, CTLA‐4 is a receptor expressed by γδ T cells and is involved in regulating their activation.^58^ To date, CTLA‐4 involvement in T‐cell checkpoint inhibition has mostly been studied in conventional T cells.^52^ Recently it was shown that Vδ2^+^ T cells with high levels of CTLA‐4 expression displayed suppressed proliferation and cytotoxic potential, further suggesting the involvement of this receptor in γδ T‐cell exhaustion and suppression.^59^

PD‐1 is another inhibitory receptor expressed by γδ T cells which likely plays an important role in immune escape by cancerous cells.^60^, ^61^ In contrast to CTLA‐4 and BTLA, PD‐1 is more involved with regulating the cytotoxic activity of γδ T cells rather than their proliferation.^52^, ^53^, ^57^ Following activation by pAgs, PD‐1 has been shown to be upregulated on the surface of γδ T cells and PD‐1 blockade has resulted in increased IFN‐γ production by γδ T cells as well as enhanced ADCC.^61^, ^62^ In leukaemic cell lines it was found that *in vitro*, the combination of pembrolizumab – an anti‐PD‐1 therapy – and Zoledronate (ZOL) was only able to significantly increase the production of IFN‐γ against one out of the four cell lines tested, which was likely a result of variable expression of PD‐1 ligand by cancer cell lines.^61^ Cancer cell lines with high levels of PD‐1 Ligand (PD‐L1) expression were also tested for the effect of anti‐PD‐L1 and anti‐PD‐1 monoclonal antibody treatment on γδ T‐cell cytotoxicity *in vitro*.^63^ It was shown that while anti‐PD‐1 therapy did not increase γδ T‐cell cytotoxicity, anti‐PD‐L1 therapy did increase the cytotoxicity of γδ T cells against cancer cells that had been pre‐treated with ZOL.^63^ These contradictory results were shown to be due to γδ T‐cell ADCC activation occurring with anti‐PD‐L1 therapy in cancer cell lines that have high levels of PD‐L1 expression.^63^

Killer cell immunoglobulin‐like receptor 2/3 (KIR2DL2/3) is another inhibitory receptor expressed by γδ T cells that has recently been demonstrated to be upregulated upon treatment with the anti‐cancer drug decitabine.^64^ KIR2DL2/3 negative γδ T cells displayed enhanced cytotoxicity to tumour cells that expressed the ligand for this inhibitory receptor when compared with KIR2DL2/3 positive γδ T cells.^64^ To date, knowledge about checkpoint inhibitors for γδ T cells remains limited but is highly relevant, particularly if immune evasion mechanisms that cancerous cells employ are to be fully understood and overcome in immunotherapy.

## Current therapies

There are two main approaches that are utilised for γδ T cell‐based immunotherapies: *in vivo* expansion using agonists to activate cells, or the adoptive transfer of *ex vivo* activated cells. *In vivo* trials utilising the expansion of γδ T cells have thus far shown limited efficacy in terms of γδ T‐cell tumour infiltration and activation, likely owing to limited knowledge about immunosuppression by the TME on γδ T cells, as well as the systemic toxicity displayed by commonly used agonists, such as n‐BPs.^26^, ^65^ Further challenges are presented by the lack of information about immune checkpoint inhibition of γδ T cells, which may be involved in inhibiting their efficacy for immunotherapy.^2^

### Therapeutic γδ T‐cell agonists

**Figure 4:**
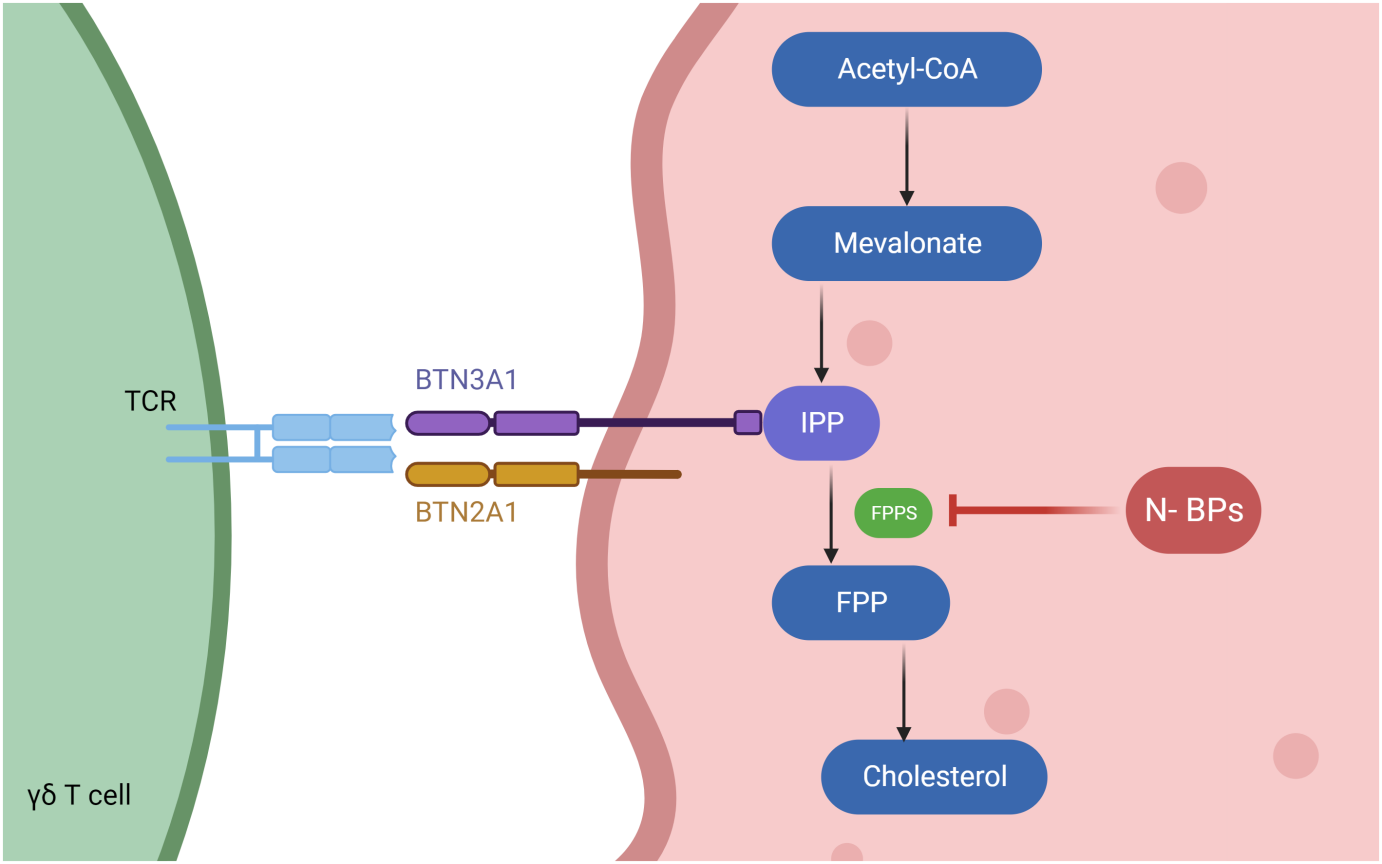
N‐BP mechanism of action whereby farnesyl pyrophosphate synthase (FPPS) is inhibited in the mevalonate pathway. FPPS is involved in the conversion of Isopentenyl Pyrophosphate (IPP) to farnesyl pyrophosphate (FPP) which can be utilised for cholesterol synthesis. Accumulation of endogenous IPP can be detected by Vδ2Vγ9 T cells due to binding of IPP to the intracellular B30.2 domain of the transmembrane protein BTN3A1. This figure is adapted from Sanz *et al*.,^107^ and was created using Biorender.

Bisphosphonates are a class of osteoporosis drugs that have been repurposed for cancer treatment due to their ability to expand populations of γδ T cells.^9^ N‐BPs were developed to improve the plasma stability of traditional bisphosphonates and target farnesyl pyrophosphate synthase (FPPS) – a critical enzyme in the mevalonate pathway, as outlined in Figure 4.^9^ Inhibition of FPPS leads to the intracellular accumulation of pAgs, such as isopentenyl pyrophosphate (IPP), which can in turn activate Vδ2Vγ9 T cells.^21^ As n‐BPs can induce higher levels of endogenous pAgs in phagocytic antigen‐presenting cells, this allows for their use as immunotherapeutic agents by initiating Vδ2Vγ9 T‐cell activation.^66^ N‐BPs such as ZOL and alendronate (ALD) are most often used as agonists for the *ex vivo* expansion of Vδ2Vγ9 T cells for adoptive cell transfer, as shown in Table 1.^50^ However, the *in vivo* use of n‐BPs in their unaltered form is limited by their suboptimal pharmacokinetics, pharmacodynamics and biodistribution. When delivered orally, which is the most common route of administration, less than 1% of n‐BPs are absorbed.^67^, ^68^ Even if this intestinal barrier is overcome, n‐BPs are small hydrophilic, charged molecules and are rapidly cleared from circulation, either through the renal system or by binding to bone.^66^ Furthermore, it has been shown that continuous dosing of n‐BPs *in vivo* can induce Vδ2Vγ9 T‐cell exhaustion, thus rendering the treatment ineffective.^50^ These issues with standard therapeutics that are used to activate γδ T cells may in part account for the limited success seen in clinical trials thus far.

**Table 1:** Clinical Vγ9Vδ2 T‐cell immunotherapeutic trials with published results

Disease	Treatment	γδ T‐cell agonist	Responses (RECIST)	Reference
Metastatic renal cell carcinoma	Innacell γδ™ (autologous *ex vivo* expanded cells)	Bromohydrin Pyrophosphate + IL‐2	n/a	96
Advanced renal cell carcinoma (*n* = 11)	Adoptive transfer	2‐Methyl‐3‐butenyl‐1‐pyrophosphate + ZOL + IL‐2	CR = 1, SD = 5, PD = 5	108
Multiple myeloma (*n* = 6)	Adoptive transfer (autologous)	ZOL + IL‐2	n/a	109
Malignant leukaemia (*n* = 4)	*In vivo*	ZOL + IL‐2	CR = 3	83
Advanced solid tumours (*n* = 18)	Adoptive transfer of autologous Vγ9Vδ2 T cells	ZOL + IL‐2	SD = 3, PD = 12, CR = 1, PR = 2	79
Non‐small cell lung cancer (*n* = 15)	Adoptive transfer of autologous Vγ9Vδ2 T cells	ZOL + IL‐2	SD = 6, PD = 6 (3 withdrawn from trial)	110
Late‐stage lung/liver cancer (*n* = 132)	Adoptive transfer of allogeneic Vγ9Vδ2 T cells	ZOL, IL‐2, IL‐15 and vitamin C	Patients who received ≥ 5 cell infusions = 18 CR = 1, PD = 2, SD = 2	72
Non‐small cell lung cancer (*n* = 25)	Adoptive transfer of Autologous Vγ9Vδ2 T cells	ZOL + IL‐2	PR = 1, SD = 6, PD = 8	73
Refractory neuroblastoma (*n* = 4)	*In vivo*	ZOL + IL‐2	n/a	80
Metastatic hormone‐refractory prostate cancer (*n* = 18)	*In vivo*	ZOL/ ZOL + IL‐2	ORR = 3	81
Metastatic renal cancer, acute myeloid leukaemia, multiple myeloma (*n* = 21)	*In vivo*	ZOL + IL‐2	ORR = 2, SD = 6, PD = 12	82

CR, complete response; n/a, not applicable; ORR, objective response rate (CR + PR); PD, progressive disease; PR, partial response; SD, stable disease; ZOL, zoledronate.

### Adoptive γδ T‐cell immunotherapy

To date, γδ T‐cell adoptive immunotherapies have yielded underwhelming results and any success has been mainly limited to haematological malignancies. The efficacy of adoptive T‐cell therapies – particularly in solid tumours – relies on the ability of γδ T cells to infiltrate tumours, yet even if this challenge is overcome, scalability still presents a major issue.^65^ In murine models, adoptive immunotherapy using *ex vivo* Vδ2Vγ9 T cells expanded by n‐BPs has shown efficacy against mesenchymal glioblastomas,^69^ osteosarcoma^70^ and epithelial ovarian cancer.^71^ More recently, adoptive Vδ2Vγ9 T‐cell immunotherapy has shown promising efficacy in clinical settings for late‐stage lung and liver cancers^72^ as well as non‐small cell lung cancer.^73^

Allogenic treatment using γδ T cells from healthy donors has proven to be clinically safe and somewhat more efficacious in the treatment of solid tumours than autologous adoptive Vδ2Vγ9 T‐cell immunotherapy, perhaps owing to an ability to overcome the immunosuppressive TME.^72^, ^73^ γδ T cells' lack of MHC restriction minimises the risk of cross‐reactivity and graft vs. host disease in patients.^2^, ^74^ Allogenic ‘off‐the‐shelf’ strategies for the treatment of tumours present enormous practical benefit in comparison to autologous therapies which are often complex, time‐consuming and costly to produce. A recent clinical trial in late‐stage lung or liver cancer patients was performed to establish the safety of allogenic Vδ2Vγ9 T‐cell treatment.^72^ Patients were injected with 4 × 10^8^ Vδ2Vγ9 T cells that had been cultured from healthy donors, once every 2–3 weeks for the first five infusions, and then either once every month or 2 months after that.^72^ The results of this study indicated that allogenic Vδ2Vγ9 T‐cell treatment is not only well‐tolerated but also efficacious, as almost all patients who received more than five infusions were shown to have significantly prolonged survival.^72^

CAR T‐cell therapies are another type of adoptive immunotherapy which typically involves introduction of a CAR transgene into conventional αβ T cells. As previously stated, there are many issues facing CAR T‐cell therapy using αβ T cells and as such, CAR γδ T‐cell therapies have been the next natural progression in the field. By using γδ T cells as the vehicle for CAR T‐cell therapies, researchers have been able to combine the specificity of CAR T cells with the advantages of γδ T cells, particularly the versatility, scalability and reduced toxicity against healthy tissue.^75^ The current state of CAR γδ T‐cell therapies has been extensively reviewed elsewhere but briefly, and further insights into γδ T‐cell checkpoint mechanisms and interaction with the TME would greatly benefit this field as well.^75^, ^76^, ^77^

A major obstacle with adoptive immunotherapies is the biodistribution of T cells following administration, as they must first reach the tumour to successfully target malignant cells.^78^ In 2011, a phase I clinical trial showed that adoptively transferred autologous γδ T cells typically migrate to the lungs, liver and spleen while also being found in some metastatic tumour sites.^79^ Recently in melanoma xenograft mouse model, tumours that were pre‐treated with a liposomal formulation of the n‐BP ALD showed decreased accumulation of γδ T cells in the liver and increased accumulation at tumour sites following adoptive treatment.^78^ This was achieved through systemic administration of liposomal n‐BPs which then accumulated at tumour sites suggesting the possibility of using passive targeting to direct administered T cells to tumours.^78^ These promising findings warrant further investigation in different cell lines and cancer models. Given the ability to safely transfer large populations of allogenic Vδ2Vγ9 T cells and effectively direct transferred cells to tumour sites, adoptive therapy remains a hopeful avenue for cancer treatment.

### *In vivo* γδ T‐cell immunotherapy

In comparison to adoptive immunotherapy, the *in vivo* expansion of γδ T cells has displayed less success, likely owing to suboptimal delivery of commonly used agonists. A combination of ZOL and IL‐2 is most often used in clinical trials to expand populations of tumour‐infiltrating and circulating γδ T cells, as detailed in Table 1.^80^, ^81^, ^82^ A phase I clinical trial in paediatric patients with refractory neuroblastoma who were shown to have significantly decreased counts of γδ T cells were treated with ZOL + IL‐2 and had on average a 3‐ to 10‐fold increase in circulating γδ T cells, restoring levels to a healthy range.^80^ A 2007 phase I clinical trial using ZOL + IL‐2 treatment in patients with hormone refractory prostate cancer showed partial remission in three patients, stable disease in five patients and, interestingly, demonstrated a statistically significant inverse correlation between serum prostate‐specific antigen levels and total number of γδ T cells after 9 months.^81^ Another pilot study in patients with advanced haematological malignancies showed on average a 68‐fold increase in administered γδ T cells after *in vivo* treatment with ZOL + IL‐2.^83^ A 2012 prospective phase I/II clinical trial in patients with either renal cell carcinoma, malignant melanoma, or AML also showed that treatment with ZOL + IL‐2 is well‐tolerated and induced partial remission in 25% of AML patients.^82^ The viability of expanding Vδ2Vγ9 T‐cell populations *in vivo* has also recently been investigated using an anti‐BTN3A agonist which has reached clinical trials.^15^ Initial results confirmed the treatment is well‐tolerated and promotes tumour infiltration of immune cells in patients with advanced‐stage solid tumours.^15^ The positive but limited outcomes in these clinical trials with *in vivo* expansion of γδ T cells may be attributed to the small sample sizes as well as these trials being a last chance treatment for advanced stage cancers. An important consideration in these trials is the fact that ZOL + IL‐2 treatment is known to induce proliferation of NK cells, hence an anti‐tumour effect from these cells cannot be completely ruled out.^82^ Given the ability to stimulate populations of γδ T cells safely and potently with minimal off‐target cytotoxicity, a targeted and efficacious *in vivo* cancer immunotherapy is within reach.

## Future directions for clinical translation

### Nanomedicine‐based approaches

**Figure 5:**
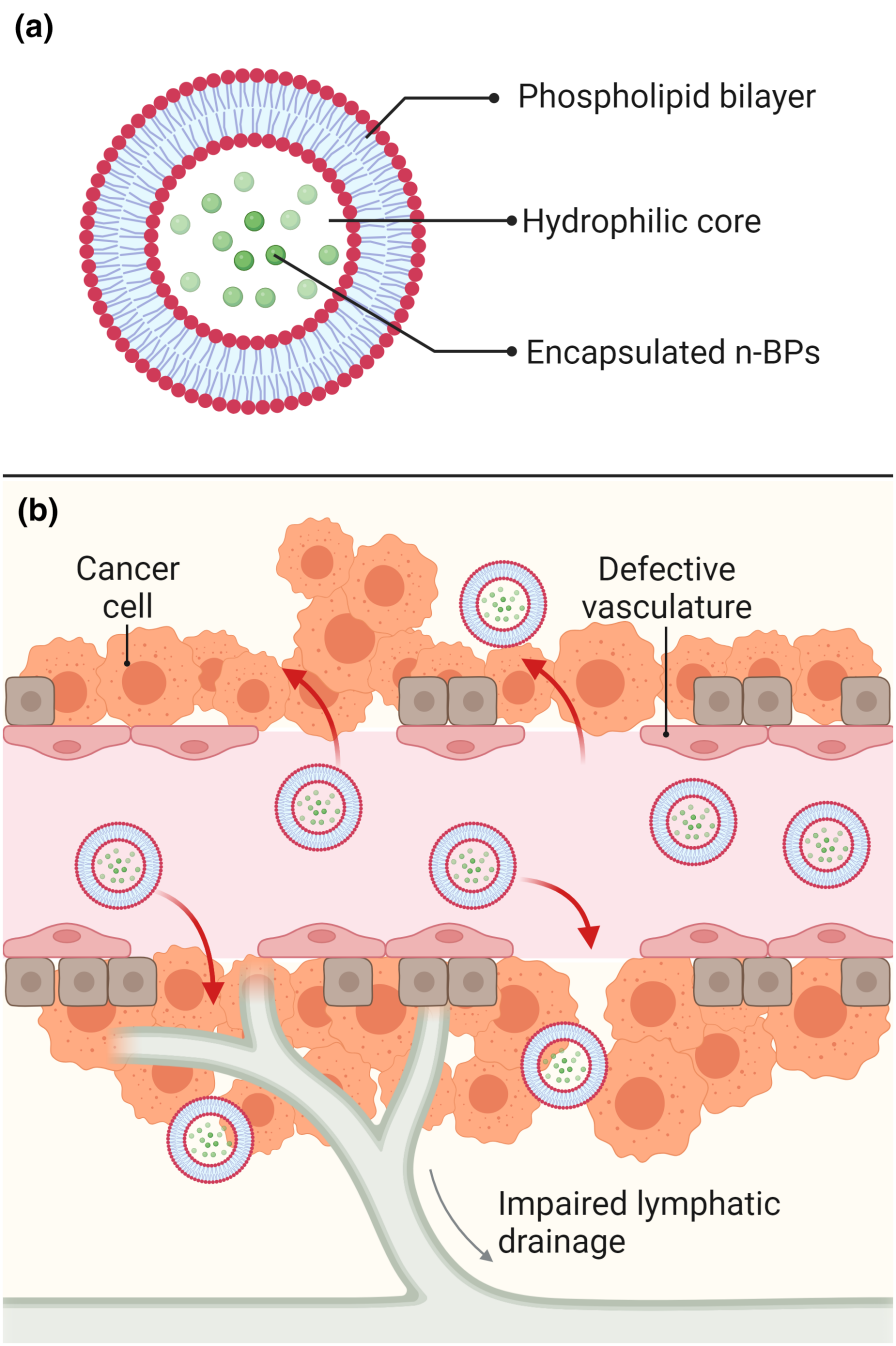
**(a)** Typical structure of simple liposomal nanocarriers which feature a phospholipid bilayer where hydrophobic drugs may be encapsulated and an aqueous core allowing for the encapsulation of hydrophilic molecules. Aminobisphosphonates (n‐BPs) are hydrophilic so are typically encapsulated within the core of the nanocarrier. **(b)** Leaky vasculature that is common to many solid tumours allows for the enhanced permeability and retention (EPR) effect meaning that small nanoparticles can extravasate from blood vessels into tumours and will not be drained by the lymph system due to impaired lymphatic drainage. This allows nanoparticles to passively accumulate at tumour sites. The figure was created using Biorender.

Nanocarriers have been used in attempts to improve the pharmacodynamic and pharmacokinetic profile of n‐BPs for use in cancer patients.^50^ While many types of nanocarriers have been explored to improve the intracellular delivery of n‐BPs, lipid‐based nanocarriers have been the most extensively utilised in the clinic due to their well‐known biocompatibility and efficacy.^9^, ^21^, ^84^ Containing both hydrophobic and hydrophilic regions, as indicated in Figure 5a, lipid‐based nanocarriers are capable of encapsulating a wide range of therapeutics and improving their solubility, cell uptake and altering their pharmacokinetic and pharmacodynamic profiles.^84^ Lipid‐based nanocarriers have also been shown to preferentially extravasate into and accumulate within tumours due to the leaky vasculature that is typical of many solid cancers, as shown in Figure 5b.^9^ These nanocarriers also offer a high degree of ‘tuneability’, allowing for surface modification with targeting ligands and other molecules, and can be used for co‐delivery of poorly bioavailable compounds.^1^, ^85^ Lipid‐coated calcium‐complexed ZOL nanoparticles have been used to prevent the premature release of ZOL and improve biodistribution to tumours.^86^, ^87^ In 2012, negatively charged liposomes containing ALD were formulated to target circulating monocytes and macrophages of the mononuclear phagocyte system (MPS).^66^ These liposomal formulations were shown to stimulate circulating γδ T cells *via* cells of the MPS while simultaneously inhibiting TAMs in a breast cancer mouse model.^66^ Anti‐tumour efficacy that has been observed in mouse models that lack γδ T cells has been suggested to be a result of endocytosis of n‐BPs by TAMs, as n‐BPs have been shown to cause the depletion of these cells.^66^, ^88^ In hepatocellular carcinoma and triple‐negative breast cancer xenograft mouse models, liposomal ZOL was shown to eliminate both M1 anti‐tumoral and M2 pro‐tumoral TAMs, favouring elimination of M2 macrophages, although this was attributed to the ratio of M1:M2 TAMs in the tumour models used.^89^, ^90^ Adoptive immunotherapy using Vγ9Vδ2 T cells activated by liposomal ALD has been demonstrated to be efficacious in the treatment of epithelial ovarian cancer in mice.^91^ Lipid‐based nanocarrier approaches have shown extensive efficacy in the activation of γδ T‐cell‐mediated lysis against cancer cells *in vitro* and in mouse models yet there are still toxicity and bioavailability issues that must be addressed to achieve clinical efficacy.

### Next generation γδ T‐cell agonists

Even with the apparent success of preclinical trials with lipid nanocarrier formulations of n‐BPs, there has been limited success in translation to human clinical trials. The lack of human clinical trials that use n‐BPs *in vivo* is most likely due to their systemic cellular toxicity attributed to inhibition of FPPS as well as rapid binding of the phosphate‐containing drugs to bone.^2^, ^12^ While lipid‐based nanocarriers have proven efficacious in improving the bioavailability of n‐BPs, there remain concerns with low cellular uptake, systemic toxicity and immunosuppression that need to be overcome before n‐BPs can be used successfully in the clinic.

As an alternative to n‐BPs, pAgs that can potently stimulate populations of γδ T cells *via* more direct mechanisms have been explored for use as immunotherapeutic agents. (E)‐4‐Hydroxy‐3‐methyl‐but‐2‐enyl pyrophosphate (HMBPP) is a small pAg molecule that is one of the most potent naturally occurring agonists of Vδ2Vγ9 T cells. HMBPP is the last intermediate in the bacterial, non‐mevalonate pathway of isoprenoid biosynthesis and can accumulate within cells which can be detected by Vδ2Vγ9 T cells.^12^, ^14^, ^92^, ^93^ Like other pAgs, HMBPP relies on an ‘inside‐out’ signalling mechanism to activate γδ T cells (see Figure 4), and so its use as a therapeutic agent is limited by its inability to passively diffuse across cell membranes.^32^ The inability of HMBPP to cross cell membranes as well as its instability in plasma means that it has limited ability to potently activate Vδ2Vγ9 T cells in its naked form, particularly in comparison to charge neutral HMBPP prodrugs that have recently been developed.^94^ In its natural form, HMBPP requires continuous dosing to achieve biologically relevant concentrations *in vivo* and for this reason, has had limited success as an immunotherapeutic agent.^93^, ^95^

Charge neutral HMBPP prodrugs, such as BrHPP (Table 1), have been synthesised in attempts to increase cellular uptake, so that this pAg may exert its intracellular effects and stimulate the proliferation of Vδ2Vγ9 T cells.^14^, ^35^, ^96^ The aim of a charge neutral HMBPP prodrug is that it may be targeted to malignant cells and more readily cross cell membranes to induce Vδ2Vγ9 T‐cell‐mediated killing.^35^ This method of *in vivo* Vδ2Vγ9 T‐cell activation relies either on the presence of tumour‐infiltrating and accessory leukocytes to aid in strong proliferation of Vδ2Vγ9 T cells within the TME or on the ability of Vδ2Vγ9 T cells to infiltrate into tumours, particularly in the case of solid tumours.^32^ An alternative strategy that has been trialled *in vitro* is incubating γδ T cells with HMBPP to stimulate their proliferation and then co‐culturing these cells with cancer cell lines.^19^ Recently, glioblastoma multiforme cells were shown to induce a Th_1_ anti‐cancer profile in Vδ2Vγ9 T cells which had been expanded using HMBPP.^19^ The *in vitro* success of HMBPP prodrugs in activating populations of Vδ2Vγ9 T cells suggests that, given improved *in vivo* pharmacodynamics and targeting to malignant cells, HMBPP may be a successful immunotherapeutic agent for tumours containing populations of γδ T cells.

Recently, a rapid expansion protocol was described for cord blood‐derived γδ T cells. In this study, lymphoblastoid cells that had been transformed with Epstein–Barr virus were used as feeder cells to stimulate the rapid expansion of γδ T cells.^97^ In comparison to peripheral blood‐derived cultures, cord blood‐derived cultures showed significantly higher fold expansion of γδ T cells which was attributed to the less differentiated nature of cells in cord blood.^98^ Cord blood γδ T cells showed the ability to elicit a broad spectrum of effector functions following expansion, which was not the case for peripheral blood γδ T cells which consist largely of the semi‐invariant Vγ9Vδ2 T‐cell subtype.^97^ The results of this study suggest that the unique phenotypic and functional profiles of Vδ2Vγ9 T cells observed in cord blood may translate to superior cancer cell targeting due to the presence of more diverse effector functions. The scalability of this method as an immunotherapy is likely limited by the accessibility of human cord blood samples. Nevertheless, this study highlights the importance of considering the phenotypic profile of the cell source as it can heavily influence efficacy following expansion.

### Combination therapy

Administration or *in vivo* expansion of γδ T cells is often insufficient on its own to overcome many of the immune evasion and suppression mechanisms that malignant cells employ. Treatments that circumvent the mechanisms that cancer cells employ to suppress and evade immune cells present a viable way to improve the efficacy of γδ T cell‐based immunotherapies. To date, this has proven difficult due to limited knowledge about γδ T‐cell inhibitory receptors as well as the specific interactions between TME chemokines and γδ T cells. Recently, melanoma spheroids were developed for use as a preclinical model to investigate the efficacy of γδ T‐cell therapy in combination with immune checkpoint inhibitors.^54^ Treatment of spheroids with γδ T cells in combination with anti‐CTLA‐4 and anti‐PD‐1 monoclonal antibodies resulted in decreased size of spheroids and displayed increased apoptosis in comparison to treatment with γδ T cells alone.^54^ Another study demonstrated that PD‐1 blockade was able to increase the expression of IFN‐γ in γδ T cells, thus enhancing their anti‐tumour capacity against AML cells, further suggesting the efficacy of γδ T‐cell immunotherapy in combination with checkpoint inhibitors.^61^

An alternative route to enhance the efficacy of γδ T‐cell immunotherapy has recently been explored that blocks the immunosuppressive interactions of the protein BTN3A1, although the mechanisms through which this occurs are not yet understood. It is suggested that BTN3A1 plays an immunosuppressive role against αβ T cells when it is not in a heterodimer conformation with BTN2A1, as has been shown to occur upon pAg‐induced activation.^15^, ^36^, ^99^ Agonistic antibodies targeting BTN3A1 that can mimic the conformational changes that occur upon binding of pAgs have been shown to restore αβ T‐cell anti‐tumour activity while simultaneously inducing γδ T‐cell‐mediated cytotoxicity.^36^ A BTN3A monoclonal antibody has recently reached phase I/IIa clinical trial stage and initial results suggest this treatment is well‐tolerated.^15^ These early results suggest the possibility of γδ T‐cell‐BTN3A1 antibody combination therapy to enhance the cytotoxic activity of administered γδ T cells.

The co‐administration of γδ T cells with activating agents also presents a viable route to improve the anti‐tumour capability of administered cells. As evidenced in Table 1, ZOL is often administered in combination with IL‐2 due to its ability to cause the proliferation of these cells.^80^, ^100^ IL‐15 secreted by DCs has been shown to enhance the cytotoxicity of γδ T cells against haematological cancer cell lines *in vitro*.^101^ Clinical trials using IL‐15 or IL‐15 derivatives have been extensively reviewed elsewhere but generally show that there are still major obstacles to be overcome preventing its use as a monotherapy.^102^ Interestingly, ascorbic acid (Vitamin C) promotes proliferation of γδ T cells and has been shown to increase their production of IFN‐γ.^103^ Synergy between vitamin C and anti‐PD‐1 has also been demonstrated, showing enhanced anti‐tumour activity in patients with B cell lymphoma, suggesting the potential benefit of a vitamin C–γδ T‐cell agonist combination therapy.^104^ The co‐administration of γδ T‐cell agonists and cytotoxicity‐enhancing cytokines and molecules could potentially aid in overcoming exhausted γδ T‐cell phenotypes, given the ability to avoid the side effects associated with high cytokine levels.

## Conclusion

Despite the apparent benefits of γδ T cells in comparison to conventional T cells, to date, γδ T‐cell clinical trials have seen limited success. This inability to translate the success seen with γδ T‐cell immunotherapy in preclinical studies to clinical trials is likely owing to several factors: the suboptimal pharmacodynamics and off‐target effects of γδ T‐cell agonists, a lack of understanding about γδ T‐cell checkpoint inhibition and tumour suppression mechanisms, as well as the direct polarising effect of the TME cytokines on γδ T cells. Furthermore, clinical trials that have been conducted to date, using either *in vivo* or *ex vivo* expanded γδ T cells, have been tested as a final treatment alternative in patients with advanced stage cancers that have been resistant to other treatments. Given these factors, a lack of efficacy in γδ T‐cell‐based immunotherapeutic clinical trials is not surprising, yet this should not deter from further exploration of this subset of immune cells in the treatment of various cancers. While checkpoint inhibition mechanisms of γδ T cells are not fully understood, these cells are not restricted by many of the mechanisms that prevent the efficacy of conventional T‐cell immunotherapies. The potential of alternative drug delivery systems – such as lipid‐based nanocarriers – to improve the pharmacodynamics of γδ T‐cell agonists as well as the use of more direct pAgs may present a viable alternative to improve upon currently used approaches. Furthermore, the use of these γδ T‐cell agonists in combination with checkpoint inhibitors or activating cytokines would likely aid in not only overcoming tumour evasion and suppression mechanisms but in enhancing the cytotoxicity of administered or expanded γδ T cells. However, before this becomes a viable option for immunotherapeutic treatment, it is necessary to learn more about the effect of the TME on γδ T‐cell polarisation and suppression. If further insight about γδ T‐cell checkpoint inhibitory receptors is gained, it would be possible to further explore combination therapy to develop an ‘off‐the‐shelf’ allogenic immunotherapeutic treatment.

## Author contributions

**Isabella A Revesz:** Writing ‐ original draft preparation; Writing – review and editing; writing – original draft preparation. **Paul Joyce:** Writing – review and editing. **Lisa M Ebert:** Writing – review and editing. **Clive A Prestidge:** Supervision; writing – review and editing.

## Conflict of interest

The authors declare no conflicts of interest.
